# Nitrous Oxide Sedation Asynchronous Curriculum for Pediatric Emergency Medicine Providers

**DOI:** 10.7759/cureus.18949

**Published:** 2021-10-21

**Authors:** Emine M Tunc, Brian Burns, Kelly Brennan, Hiromi Yoshida, Rebekah Burns

**Affiliations:** 1 Pediatric Emergency Medicine, University of Washington School of Medicine, Seattle, USA; 2 Pediatric Emergency Medicine, Seattle Children's Hospital, Seattle, USA

**Keywords:** qr code, pediatric emergency medicine, asynchronous curriculum, procedural sedation, nitrous oxide

## Abstract

This technical report describes a nitrous oxide sedation training curriculum for pediatric emergency medicine providers. This curriculum was used during the novel coronavirus disease 2019 (COVID-19) pandemic where in-person classroom training was significantly limited. We demonstrate a model for concept and equipment learning with video-guided self-practice in place of in-person training with a facilitator. A similar model can be utilized for other equipment or concept training.

## Introduction

Sedation is commonly used in pediatric emergency departments (PEDs) for various procedures to alleviate pain and anxiety. Common pediatric procedures include laceration repair, fracture reduction, lumbar puncture, and incision and drainage [1]. Different levels of sedation may be needed depending on the procedure, patient’s age, pre-procedure anxiety, and developmental history. The level of sedation can be classified as mild, moderate, deep sedation, and general anesthesia based on the patient’s level of responsiveness to stimuli. Sedation is a continuum, and a patient can move from one level to another during a procedure [2].

Nitrous oxide (NO) is a colorless gas that provides minimal to moderate sedation. Advantages of NO include (1) lack of pre-sedation fasting requirements, (2) noninvasive administration, (3) ability to titrate the level of sedation (i.e. patient can be sedated more during the painful parts of the procedure), (4) fast onset and offset, (5) short post-sedation observation period, and (6) infrequent cardiopulmonary adverse events. The disadvantages of NO are that its use is limited to minimal to moderate sedation, it has only minimal analgesic effects, and its administration is labor-intensive [3-5].

Our PED began using NO in 2017, and it was administered exclusively by the nursing staff [6]. In an effort to increase the availability of NO sedation, training on its administration was then extended to pediatric emergency medicine (PEM) providers. Due to social distancing measures during the coronavirus disease 2019 (COVID-19) pandemic, in-person training was significantly limited in our institution resembling many other institutions globally [7,8]. Thus, we designed an asynchronous curriculum to limit in-person contact.

This article was previously presented as a poster at the University of Washington CLIME Together Symposium on June 11th, 2021, and the Annual International Telesimulation in Healthcare Conference on September 23rd, 2021.

## Technical report

Participants

The curriculum was designed for the PEM providers including fellows, advanced practice providers, and attendings. All providers had prior experience with minimal to moderate sedation and managing sedation adverse events (i.e. respiratory depression). Providers had basic knowledge on patient selection, indications, and contraindications for NO sedation prior to the training. Only seven of the 60 providers had prior experience with NO administration.

Setting and equipment

Our institution had been using nurse-administered NO for sedation in the PED. In this training model, a hands-on demonstration by one of the NO instructors was done to train new nurses. Novice users were required to administer NO under supervision at least once to obtain credentials to administer independently. The PEM providers planned and ordered NO sedation but did not participate in the administration.

There are a variety of nitrous oxide delivery systems commercially available, but the principles of the delivery systems are the same. A delivery system includes an oxygen source, nitrous oxide source, scavenging system, patient interface (face mask, nasal prongs, or demand valve), circuit, oxygen flow meter, and nitrous oxide titration meter [9]. For the purpose of this curriculum, Porter Sentry Sedate HP (Hatfield, PA) portable nitrous oxide delivery system was used.

Curriculum

The asynchronous curriculum consisted of an online module and video-guided self-practice. The online module was composed of a slide show reviewing NO mechanism of action, indications and contraindications, and complications, and troubleshooting video-guided self-practice included eight videos uploaded to a YouTube (San Mateo, CA) channel (Videos 1-8).

**Video 1:** Gathering supplies

**Video 2:** Review of the nitrous oxide delivery system and apparatus

**Video 3:** Operating nitrous oxide tank

**Video 4:** Five-step safety check

**Video 5:** Starting the sedation

**Video 6:** Titrating nitrous oxide level

**Video 7:** Ending the sedation

**Video 8:** Clean up

The videos were recorded by one of the authors (B.B.) demonstrating the use of NO step by step. The individual steps included: (1) gathering supplies, (2) review of the NO delivery system and apparatus, (3) operating nitrous oxide tank, (4) five-step safety check, (5) starting the sedation, (6) titrating nitrous oxide level, (7) ending the sedation, and (8) clean up. A novice user took about 30 minutes to complete this practice. Video-guided self-practice essentially replaced hands-on training with an instructor.

**Figure 1:**
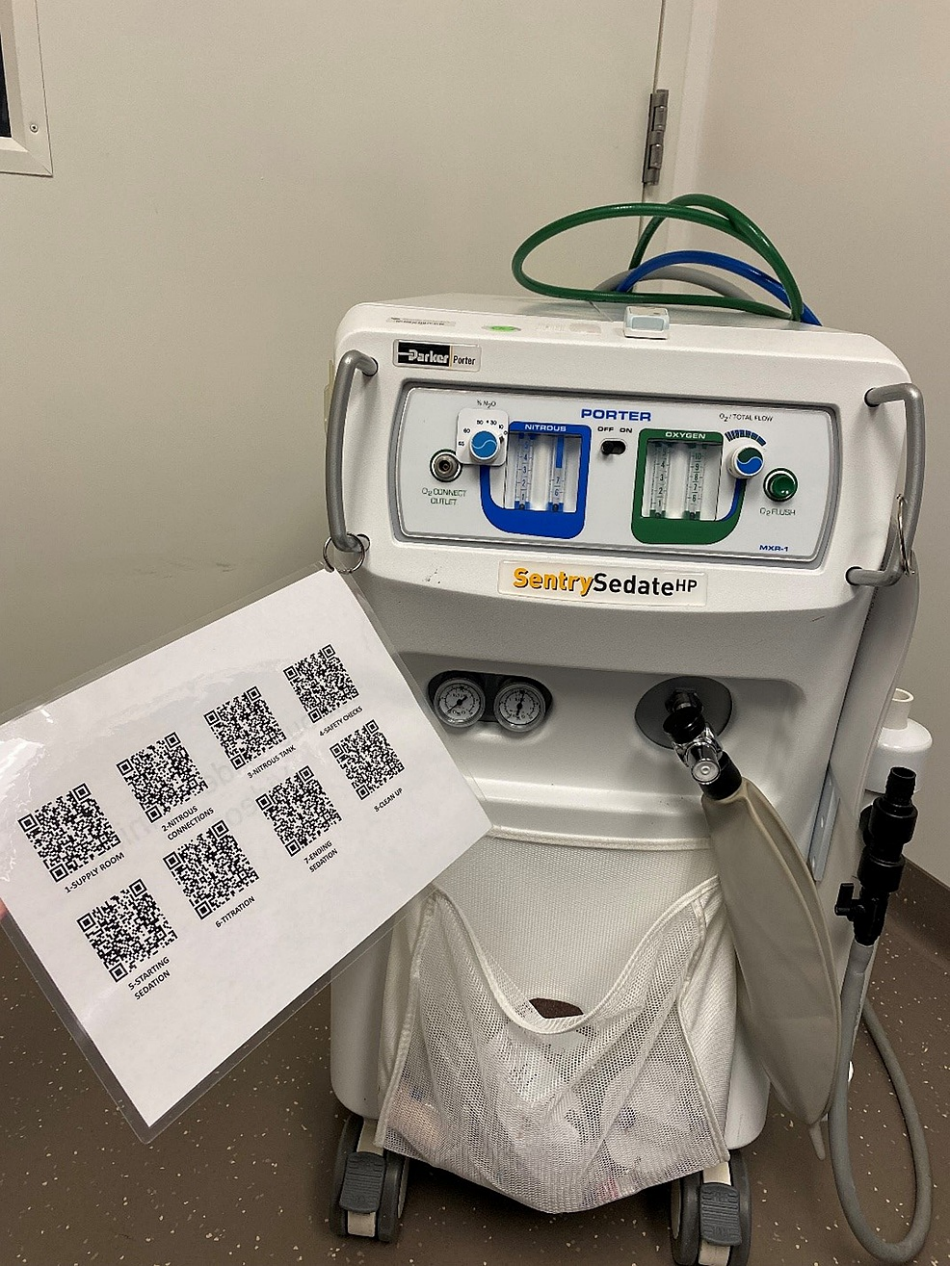
Nitrous oxide machine and QR codes with the links to the training videos QR, quick response.

We used an innovative approach for accessing the training videos by quick response (QR) codes. The QR codes were available on the NO machine for review and practice at any time and, most importantly, just prior to providing sedation (Figure 1). To reinforce the teaching and provide an opportunity to ask questions, we held a two-hour online synchronous lecture where instructors (E.T. and B.B.) reviewed the slide show (described above) and demonstrated how to use the machine step by step. This lecture was held two weeks after the online module and videos were made available.

The novice users who completed the online module and video-guided self-practice were asked to administer NO with supervision at least once to obtain credentials to administer independently. If a provider had prior experience of NO administration, they were exempted from this requirement and could administer independently after completing the curriculum.

Assessment

After completing the online module, the participants took a brief test (Appendix 1). A threshold of 80% was required to pass. After completing the video-guided self-practice, participants filled out a brief survey to indicate completion. The survey link was provided along with the links to the videos. A QR code was created for the survey link and training videos. These were available on the NO machine. Novice users completed an additional survey to indicate the date and the name of the observing supervisor after completing NO sedation under supervision.

A third voluntary survey was sent to the participants via email to assess the curriculum. Twenty-three providers completed the online module and video-guided self-practice. Twenty-two providers responded to the voluntary curriculum assessment survey, which was sent separately via email. The survey used the Likert scale (strongly agree, agree, neutral, disagree, and strongly disagree) and asked if the “curriculum was easy to complete at my own pace,” “curriculum was effective,” “QR codes made the training more accessible,” and “I can administer nitrous oxide independently.” Responses of “strongly agree” and “agree” were considered as positive responses. Of the participants, 72% had never administered NO before. All of the participants reported that the curriculum was easy to complete at their own pace, and 100% found the curriculum effective. Of the participants, 86% found QR codes helpful for both the initial and just-in-time review. Confidence in administering NO increased from 18% to 77%. Of the participants, 30% found the live lecture redundant, 30% were neutral, and 35% found it necessary.

## Discussion

This curriculum has several benefits. The video-guided self-practice while using the equipment allows the learner to practice at their own time and pace. This method also allows learners to practice more than once. QR codes provide easy access to the online curriculum content. The limitation of the curriculum is the lack of in-person instructors and the ability to ask questions and receive feedback. Learners were encouraged to note their questions during the self-guided practice and ask an instructor later.

This curriculum can be used to train physicians, fellows, advanced practice providers, and nurses for NO sedation. Instructional videos for other nitrous oxide delivery systems can be created if needed.

Equipment training often requires tactile feedback where the trainee performs a hands-on review of the equipment while manipulating the actual buttons, knobs, and tubing. When available, an instructor is helpful to demonstrate the use of the equipment and answer questions. That approach requires instructor availability and may not be feasible when social distancing measures are required. Here, we described a model to achieve equipment training with slide-based education and video-guided self-practice that is asynchronous. A similar curriculum can be created for new equipment or concept training [10-14].

## Conclusions

This technical report describes an asynchronous curriculum for nitrous oxide sedation training. This can be completed at a convenient time for learners and done at their own pace. With the curriculum, the provider can practice repeatedly and gain confidence in administrating NO. A similar model can be used to train providers for new equipment or concepts.

## Appendices

Post online module test

1) What actions does nitrous oxide provide?

A. Relaxation

B. Mild pain relief

C. Euphoria

D. All of the above

2) In addition to oxygen administration, what is absolutely essential to have during nitrous oxide administration?

A. Albuterol

B. Scavenging

C. Scented face mask

D. Music playing

3) To support the patient to be guided into a relaxed state, what strategies can be used?

A. One voice

B. Allowing three to five minutes for the onset of action before the procedure starts

C. Calm environment

D. All of the above

4) Nitrous oxide is considered minimal sedation, but if given with an opioid or benzodiazepine, it is considered moderate sedation.

A. True

B. False

5) Contraindications for nitrous oxide are all the following except:

A. Trapped gas such as bowel obstruction and pneumothorax

B. First trimester pregnancy

C. Laceration

D. B12 deficiency

Answer key

1. D

2. B

3. D

4. A

5. C
